# Decoding bladder state from pudendal intraneural signals in pigs

**DOI:** 10.1063/5.0156484

**Published:** 2023-10-05

**Authors:** A. Giannotti, S. Lo Vecchio, S. Musco, L. Pollina, F. Vallone, I. Strauss, V. Paggi, F. Bernini, K. Gabisonia, L. Carlucci, C. Lenzi, A. Pirone, E. Giannessi, V. Miragliotta, S. Lacour, G. Del Popolo, S. Moccia, S. Micera

**Affiliations:** 1 The BioRobotics Institute and Department of Excellence in Robotics and AI, Scuola Superiore Sant'Anna, Pisa, Italy; 2 Neuro-Urology Department, Careggi University Hospital, Firenze, Italy; 3 Bertarelli Foundation Chair in Translational NeuroEngineering, Neuro-X Institute, École Polytechnique Fédérale de Lausanne, Lausanne, Switzerland; 4 Laboratory for Biomedical Microtechnology, Department of Microsystems Engineering–IMTEK, IMBIT//NeuroProbes BrainLinks-BrainTools Cluster of Excellence, University of Freiburg, Freiburg, Germany; 5 Bertarelli Foundation Chair in Microengineering and Bioengineering, Neuro-X Institute, École Polytechnique Fédérale de Lausanne, Lausanne, Switzerland; 6 BioMedLab, Scuola Superiore Sant'Anna, Pisa, Italy; 7 Department of Veterinary Sciences, University of Pisa, Pisa, Italy

## Abstract

Neuroprosthetic devices used for the treatment of lower urinary tract dysfunction, such as incontinence or urinary retention, apply a pre-set continuous, open-loop stimulation paradigm, which can cause voiding dysfunctions due to neural adaptation. In the literature, conditional, closed-loop stimulation paradigms have been shown to increase bladder capacity and voiding efficacy compared to continuous stimulation. Current limitations to the implementation of the closed-loop stimulation paradigm include the lack of robust and real-time decoding strategies for the bladder fullness state. We recorded intraneural pudendal nerve signals in five anesthetized pigs. Three bladder-filling states, corresponding to empty, full, and micturition, were decoded using the Random Forest classifier. The decoding algorithm showed a mean balanced accuracy above 86.67% among the three classes for all five animals. Our approach could represent an important step toward the implementation of an adaptive real-time closed-loop stimulation protocol for pudendal nerve modulation, paving the way for the design of an assisted-as-needed neuroprosthesis.

## I. INTRODUCTION

The lower urinary tract (LUT) is responsible for the storage and elimination of urine, thanks to the coordinated and antagonist activity of the bladder, bladder neck, and urethral rabdomiosphincter.^1^ The functional activity of these smooth and striated muscles is regulated by complex neural mechanisms involving both the central and peripheral nervous system.^2^ Neurological diseases such as spinal cord injuries, multiple sclerosis, or cerebral lesions (e.g., Parkinson's disease, brain injury, …) may cause neurogenic LUT dysfunctions (LUTD). Such dysfunctions include urinary incontinence and/or incomplete bladder emptying^2,3^ and may profoundly affect the social and emotional well-being of the affected patients.^4^

Neuroprosthetic devices have been adopted in the last few years to restore normal micturition or relieve LUT symptoms.^5^ These devices use a tined lead electrode placed beside the nerve to continuously or intermittently inject an electrical current.^2^ They are used for sacral nerve stimulation (SNS) and tibial nerve stimulation (TNS), as well as used for the treatment of overactive bladder, urinary retention, and fecal incontinence.^6,7^ The long-term efficacy of these treatments is still highly variable: while the success rate (>50% reduction in symptoms) of a SNS chronic implant ranges from 29% to 76%, the success rate of TNS is in the range of 54%–59%.^8^

The Food and Drug Administration approved neuroprosthetic devices using a continuous, open-loop stimulation paradigm where the electrical stimulation does not consider the bladder-filling state. As a result, there is the possibility of neural adaptation to stimulation, which causes a decrease in voiding efficacy.^9^ Researchers are comparing the effects of conditional, closed-loop stimulation paradigm to those of open-loop stimulation both in animal models and in humans, confirming the need to move from neuroprosthesis with predefined activation to assisted-as-needed neuroprosthesis.^10–12^ State-dependent pudendal nerve stimulation has been shown to increase both bladder capacity and voiding efficacy in rats by using cuff electrodes compared to the continuous stimulation paradigm.^9^ Challenges in implementing closed-loop stimulation include predicting the state of bladder filling and determining the target nerve and electrical stimulation parameters, making clinical protocols for LUTD currently absent.^13,14^

To take a step forward in the design of a closed-loop system for the treatment of LUTD, in this study, we designed an algorithm for decoding bladder-filling states from ElectroNeuroGraphic (ENG) signals of the pudendal nerve. Our decoding strategy relies on the use of Machine Learning (ML) to tackle the variability of ENG signals. Our approach may enable a future implementation of a fully closed-loop neuroprosthesis, which would allow us to predict bladder-filling states and apply nerve stimulation when necessary, without the need to implant additional pressure sensors, which are prone to erosion and sensor drift.^14^

We decided to focus on the pudendal nerve because of the promising results for detrusor contraction detection using information carried by afferent neural pathways.^14^ Furthermore, pudendal nerve stimulation has been investigated as an alternative target for neuromodulation systems to restore genitourinary function, showing promising results in terms of efficacy and bladder control compared to currently established neurostimulation therapies.^15–17^

Our ENG signal acquisition strategy relied on penetrating transversal intrafascicular multichannel electrodes (TIMEs) to acquire pudendal ENG signals. TIMEs demonstrated good stability and selectivity with proven long-term efficacy, enabling increasing the signal-to-noise ratio of the ENG recordings.^18^ Our recent studies have shown how ML algorithms can successfully allow us to decode different types of information from TIME neural recordings acquired from median, ulnar, and vagus nerve.^19–21^

**FIG. 1.**
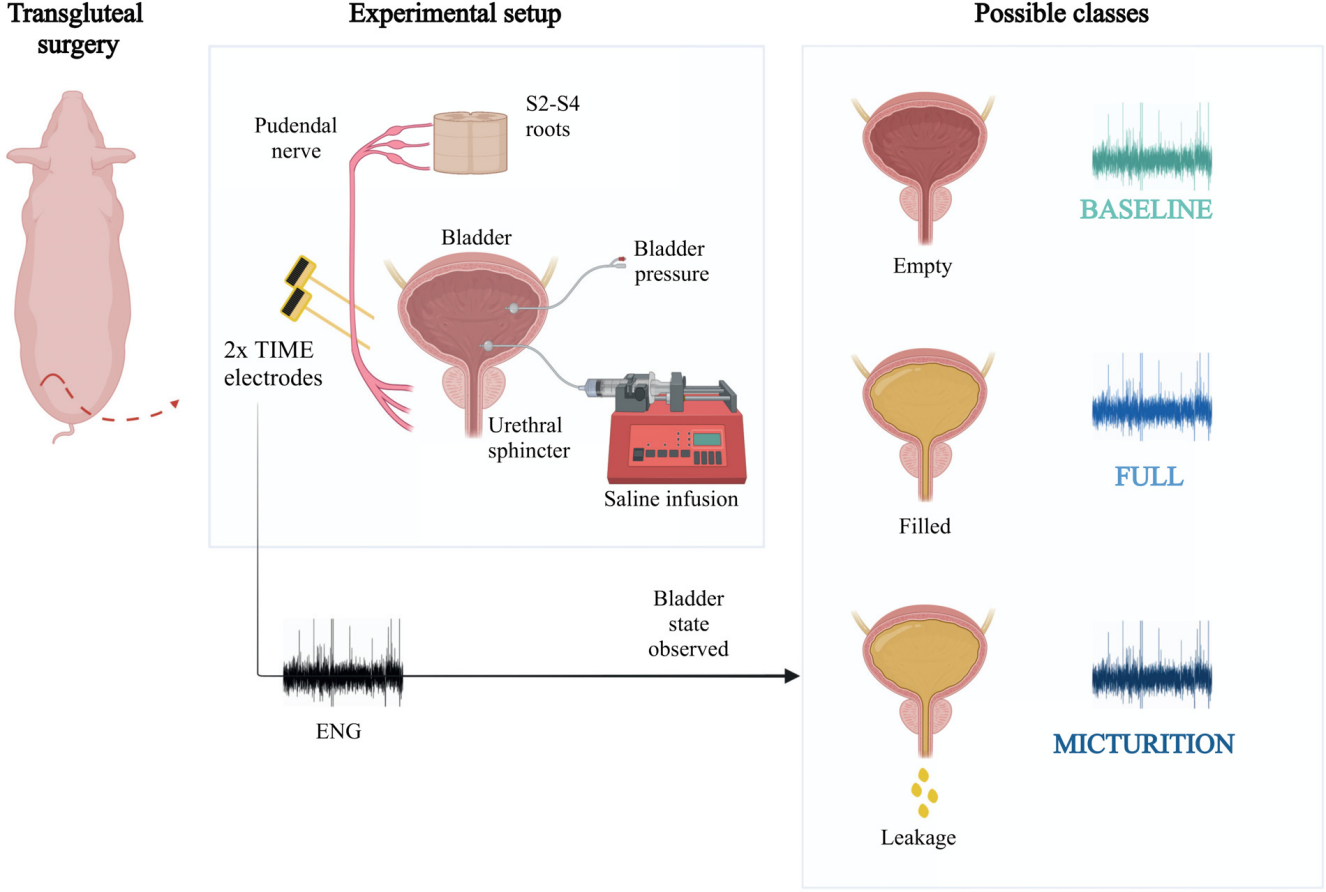
ENG signal acquisition implanting two TIME electrodes in the pudendal nerve through transgluteal surgery. Signals, which refer to bladder emptiness, fullness, or leakage state, are classified by our decoding algorithm as baseline, full, and micturition, respectively.

We collected ENG signals from the pudendal nerve of anesthetized pigs during bladder filling to characterize the various physiological conditions of the bladder, starting from empty to full bladder and micturition (Fig. 1). A large animal model such as the pig allowed us to work with a LUT anatomy closer to that of humans, facilitating the translation from preclinical to clinical trials of surgical technique and analysis of acquired ENG signals.

To summarize, the main contributions of this study can be listed as follows:

• Collecting the first *in vivo* dataset of pudendal ENG signals in pigs related to bladder-filling by using intrafascicular TIMEs;
• Designing a ML algorithm able to automatically decode three bladder-filling states, corresponding to emptiness, fullness, and micturition, from ENG pudendal nerve signals.

## II. RESULTS

A qualitative histological analysis of the pudendal nerve explanted from pigs was performed to customize the design of TIMEs implanted to acquire ENG signals during bladder filling, as described in Sec. II A. We conducted the experiment of simultaneous bladder infusion and recording on large animal models, implanting the custom-designed neural interface. The ENG pudendal nerve signals were classified through different ML models, as described in Sec. II B, to predict bladder state between the three different conditions listed in Sec. I.

### A. Electrode design and implantation

**FIG. 2.**
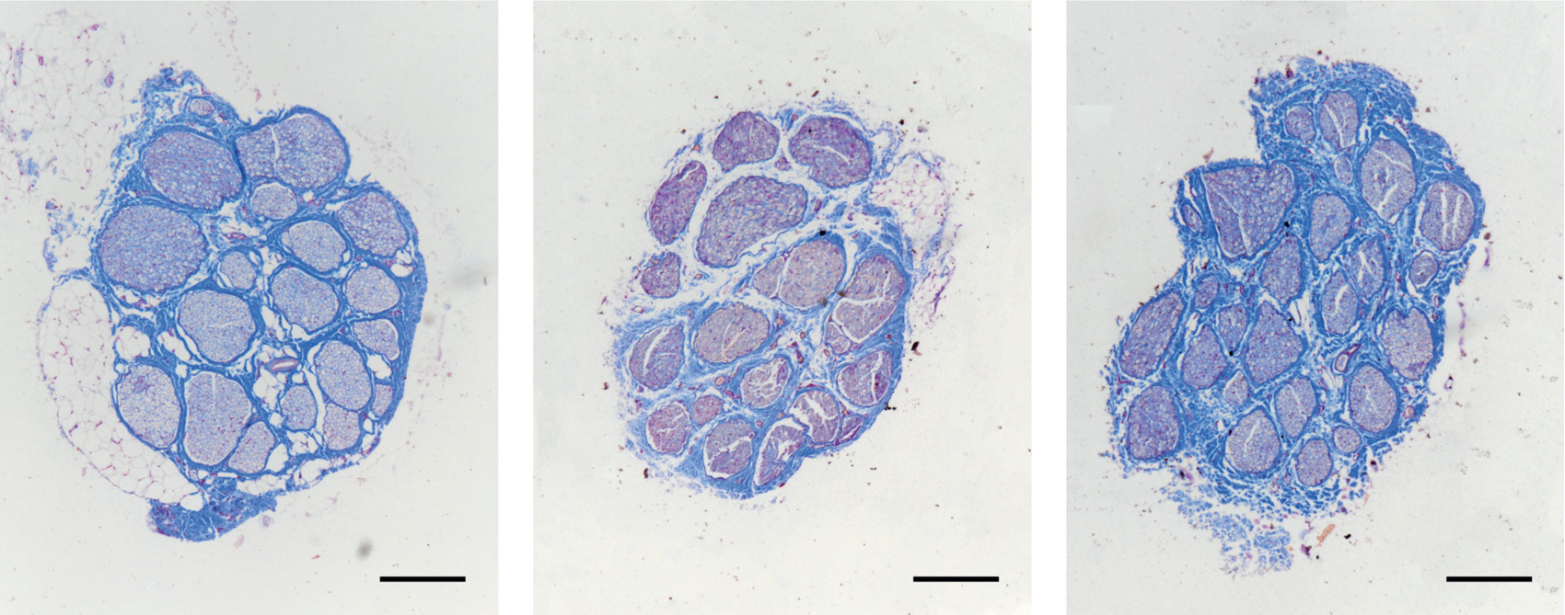
Histological images of the porcine pudendal nerve stained with Mallory's trichrome protocol belonging to three of the 16 different pudendal nerve specimens described in Sec. V A. Scale bar 200 *μ*m.

**FIG. 3.**
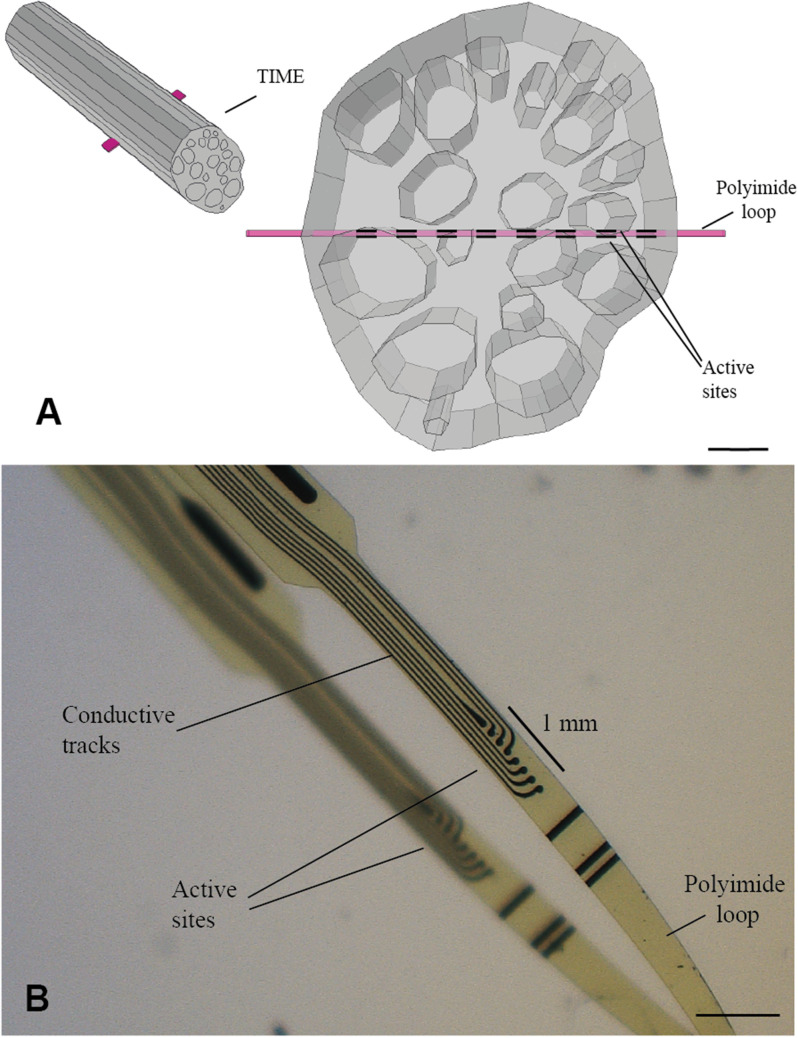
(a) Three-dimensional model of the pig pudendal nerve generated from a histological pudendal nerve section with a TIME electrode implanted. The right view of the three-dimensional model shows the customized position of the ASs within the pudendal nerve cross-section. Scale bar 200 *μ*m. (b) TIME electrode used for pudendal nerve recording experiments. Scale bar 200 *μ*m.

Sixteen histological sections, stained with Mallory's trichrome protocol and explanted from pudendal nerve samples during previous studies on pigs, were analyzed. Figure 2 shows histological sections of the porcine pudendal nerve (Fig. 1S of the supplementary material also shows different sections belonging to the same nerve). The average pudendal nerve diameter was 961 *μ*m (std ± 88). The average number of fascicles was 22 (std ± 5). The average smaller diameter fascicle was 66 *μ*m (std ± 13). The electrode design specifications were selected according to the anatomical characteristics found. Figure 3(a) shows a three-dimensional model (COMSOL Multiphysics 5.3, Comsol Inc., SE) of the pig pudendal nerve obtained from a histological image, implanted with an electrode having a geometrical design matching with dimensions of the nerve under investigation. More specifically, the maximum distance between active sites (ASs) was chosen to be 1 mm based on the diameter of the nerve, while the diameter of the ASs was chosen to be 60 *μ*m in order to have an AS that could selectively target smaller fascicles. Figure 3(b) shows the fabricated electrode.

**FIG. 4.**
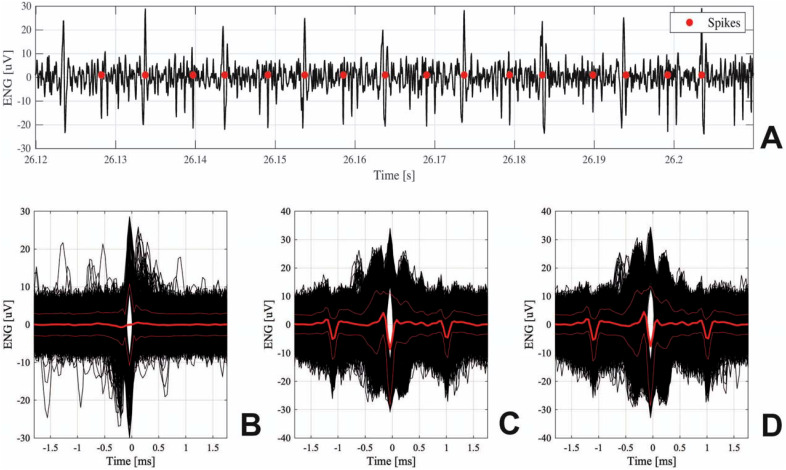
(a) Spike detection (red dots) after bandpass filtering, with a refractory period of 4 ms. Superimposed detected spikes (bold red line represents mean, and thin red lines represent standard deviation) from (b) baseline; (c) full; and (d) micturition states, respectively.

**FIG. 5.**
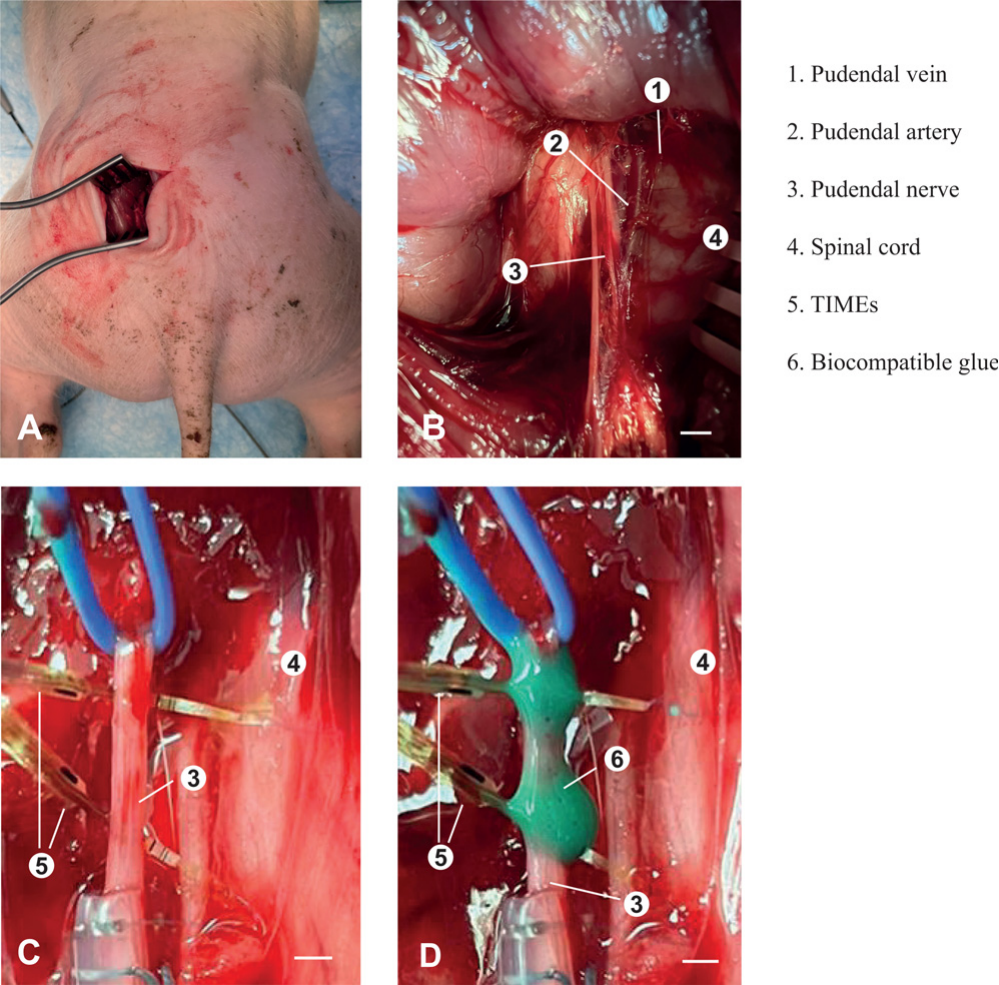
(a) Transgluteal surgical approach to expose the pudendal nerve in pigs. (b) Close-up of pudendal nerve exposure. Scale bar 4 mm. (c) TIMEs implanted within the pudendal nerve. Scale bar 1.5 mm. (d) TIMEs implanted and stabilized in position with biocompatible glue. Scale bar 1 mm.

Two TIMEs with 16 ASs were successfully implanted in the five pigs, named p1, p2, p3, p4, and p5, to record neural activity related to the bladder-filling states. Figure 4(a) shows spikes detected from filtered ENG signal recorded during the micturition phase, while Figs. 4(b)–4(d) show superimposed detected spikes corresponding to emptiness, fullness, and micturition bladder-states, respectively. In the supplementary material are also reported raw signals of pudendal nerve ENG activity in empty conditions from eight active sites of the TIME (Fig. 2S, supplementary material), and different shapes of the spikes detected from ENG signals acquired during the three bladder states (Fig. 3S, supplementary material). A transgluteal approach was used to expose surgically the pudendal nerve, as shown in Figs. 5(a) and 5(b). Figures 5(c) and 5(d) show the two electrodes at the end of the implantation procedure and after the application of biocompatible glue which ensures implant stability, respectively.

### B. Decoding algorithm

**FIG. 6.**
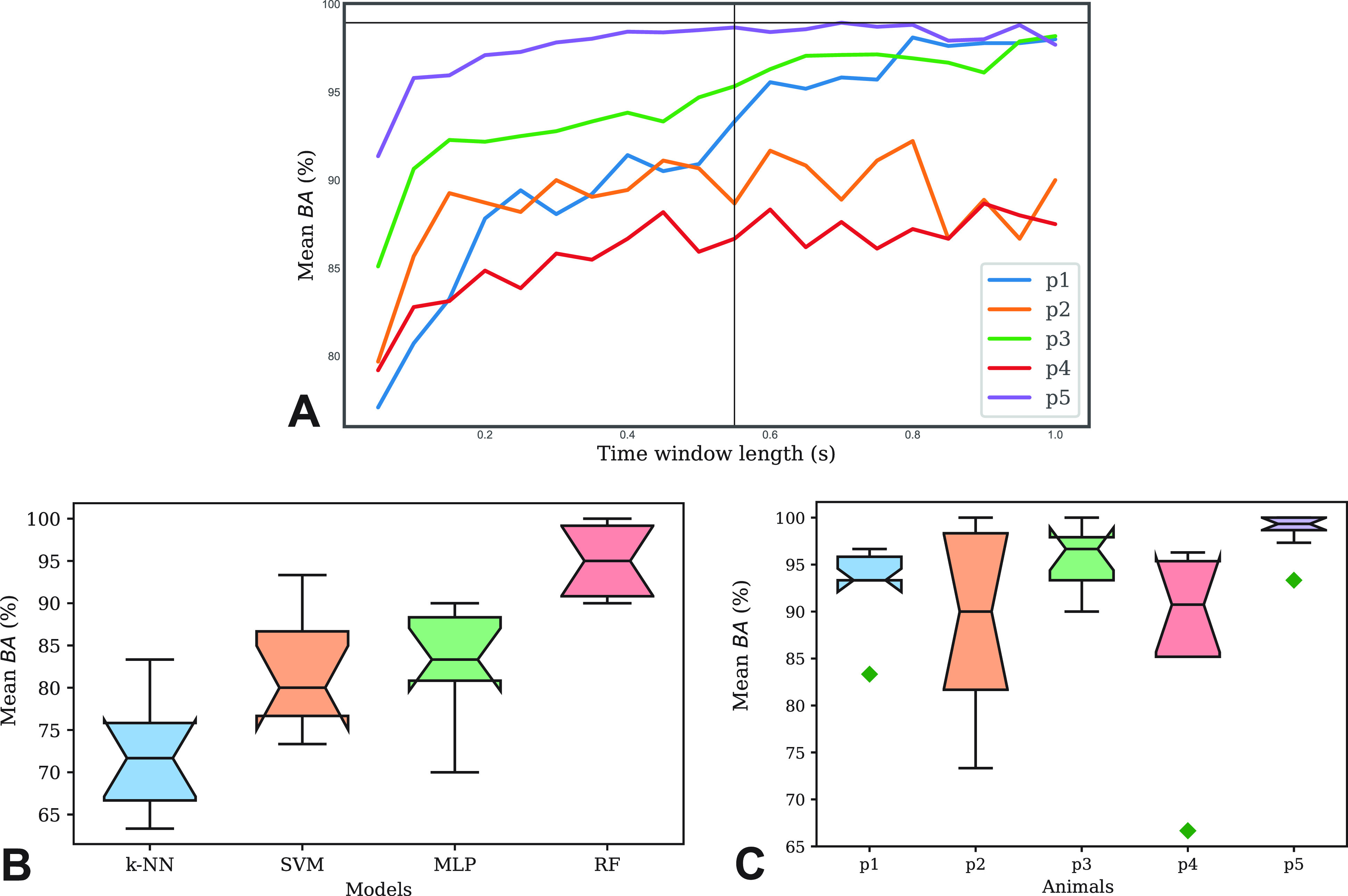
(a) Mean test *BA* for each animal model (p1–p5) at varying time window length. (b) Average *BA* in tested Classifiers. The colored boxes' lower and upper extremities represent the first and third quartiles delimitating the interquartile range, the whiskers show the outliers, the lines in the middle of the boxes represent the medians, and the notches in correspondence of them represent their confidence interval. (c) Boxplot comparison between animals in terms of *BA.*

For each pig, each channel recording was divided into time windows, and nine handcrafted features were extracted from each window: mean, variance, skewness, kurtosis, maximum amplitude, wavelength, maximum, amplitude, and power. Window length and overlap ratio were tuned, performing Random Forest (RF) classifications for each pig while varying these two parameters. Figure 6(a) shows the mean Balanced Accuracy (*BA*) values across all test folders obtained following ten-fold cross-validation for each animal model independently. A trade-off between the highest *BA* and smallest window length for possible further real-time application has been considered. A time window length equal to 0.55 s and an overlap to increase the numerical size of the training set equal to 0.11 s were chosen for the following steps.

Figure 6(b) shows the mean *BA* between the ten test folds for RF, as well as Support Vector Machine (SVM), k-Nearest Neighbors (k-NN), and Multi-Layer Perceptron (MLP), for one animal model. Results confirmed all four models to be good candidates for real-time applications, considering their computational performance. RF outperformed the other classifiers with a mean *BA* equal to 95.00%, with an interquartile range equal to 8.33. The RF was used in all subsequent studies, enlarging the database to all animal data available.

**FIG. 7.**
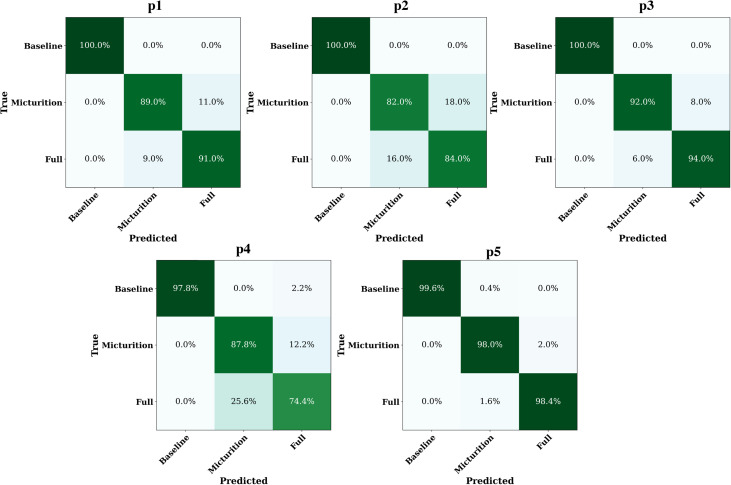
Confusion matrices obtained from RF models output for each pig (noted as p1, p2, p3, p4, and p5).

RF classification results were further investigated by comparing single class prediction in terms of average test *BA* across all animals. Figure 6(c) shows the *BA* mean and standard deviation for each animal. Mean *BA* values are all above 86.67%. Confusion matrices are shown in Fig. 7. Correct predictions for the baseline class were above 97.80%, while full and micturition classes show satisfying prediction accuracy. Full state has shown an average successful prediction equal to 88.36%, while for the micturition state, the average is equal to 89.76%.

**FIG. 8.**
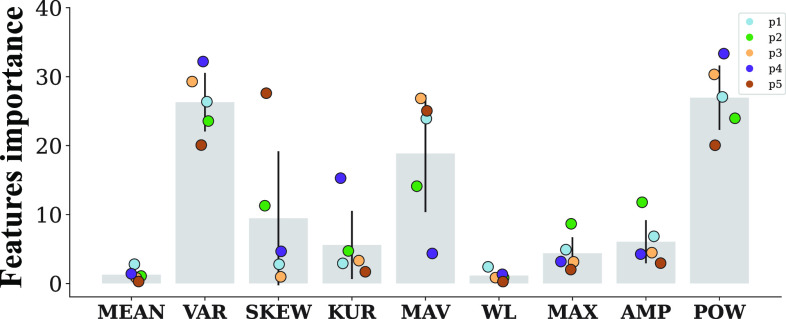
Average feature importance assessed with the Gini importance index for all pigs (noted as p1, p2, p3, p4, and p5). The features computed from signals' time windows were mean (MEAN), variance (VAR), skewness (SKEW), kurtosis (KUR), maximum amplitude (MAV), wavelength (WL), maximum (MAX), amplitude (AMP), and power (POW).

Feature importance was compared between the five animals. Each value relative to a single animal has been obtained as the average of all ASs' results. In order to evaluate the feature importance for each recording site, the Gini index was computed. Feature importance is inversely proportional to the decrease in its impurity, quantified by the Gini index. As shown in Fig. 8, variance, power, mean absolute value, and skewness showed to be the features with the highest decrease in impurity, hence the highest importance, across the animals analyzed. We also observed that ASs contributed unequally to the final *BA*, even though the specific channels of major importance appeared to be strongly dependent on the animal.

## III. DISCUSSIONS

Previous literature studies highlighted the need for a neural interface that can selectively detect and differentiate different genitourinary functions to improve the accuracy of bladder monitoring and the effectiveness of a closed-loop stimulation system.^22^ In previous work conducted on cats, the ENG pudendal nerve trunk signals collected through a tripolar cuff was explored for hyper-reflexive bladder contraction detection through cumulative sum algorithms.^23^ The results obtained show a detection delay above 1 s, which could be improved for real-time applications. In a previous work, another approach on the same target nerve was investigated,^24^ applying a nonlinear model algorithm based on a neural network for bladder pressure estimation. Despite the high robustness of prediction, its high complexity could be critical in terms of computational cost for closed-loop applications. The results of this study showed that ML algorithms can be used to automatically decode three bladder-filling states from intraneural ENG pudendal nerve signals in a large animal model.

This work reports the first *in vivo* dataset of pudendal ENG signals in pigs related to bladder filling using intrafascicular TIMEs. As shown in Sec. II A, TIMEs were used to acquire selective pudendal ENG recordings. In order to select the optimal implantation position in the nerve, histological analysis of the target nerve allowed the distribution of ASs within the whole cross-sectional area. Since the pudendal nerve is composed of fibers of different natures, pudendal nerve stimulation is a powerful target to treat multiple pelvic functions using the here proposed ML decoding algorithm. Depending on the stimulation frequency of efferent fibers, the bladder detrusor can be relaxed to promote continence or excited to promote micturition.^25^ The afferent fibers sense urine flowing through the urethra regulating through neural reflexes the detrusor muscle activity, thus their activity can be used as an input of the decoding algorithm here proposed.^1^

Each model is animal-specific, hence training and testing are performed for each pig separately. Considering the models tested, the RF showed better results than other classifiers in terms of mean testing *BA*. The states characterizing bladder condition from empty to progressive filling are well-predicted with an overall successful prediction of above 86%. Feature importance reflects expectations, showing a common signal behavior to results obtained in previous work.^20^ Mean *BA* results confirm, as suggested in previous studies^20^ for the classification during cardiac and respiratory challenges, that this approach can be used to tackle data scarcity to classify bladder state with ML algorithm.

Collected data showed a mean *BA* above 80% in all animals. However, it was noticed both during the experimental phase and from the spectral power density analysis that specific animals' recordings were affected by distributed noise that worsens classification outcomes, as evident looking at the p2 *BA* boxplot in Fig. 8. Minor changes in the implantation site, operating room conditions, number of operators present, and instrumentation noise were revealed to impose a significant effect on ENG recordings in specific cases. During the pre-processing phase, data were filtered by a Butterworth bandpass filter, without any additional pre-processing for enhancing signal-to-noise ratio. Improvements to recording experiments' instrumentation could be refined by developing a lightweight pre-amplifier system to be attached in close proximity to the intrafascicular electrode implanted. In addition, a proper filter to be applied for signal refinement could be investigated.

Feature comparison between animals highlighted that variance, maximum amplitude, and power carry the most important contribution in decreasing the trees' impurity for the majority of the animals analyzed, confirming recent results.^25^ Furthermore, our results seem to confirm that ASs did not perform equally. This underlines the importance of a multichannel intrafascicular interface recording but also the presence of an implantation site that could guarantee afferent fibers detection.

Most frequent misclassifications were noticed between full and micturition classes. However, this was expected since the two states occur physiologically in sequence with a strongly nonlinear detrusor pressure change. Therefore, there is a chance that the pudendal activity experiences a period of increasing adaptation during advancing bladder filling. The prediction error was most noticeable in experiments most affected by high-frequency noise or electronic instrumentation noise. A multi-animal trained model would be a powerful tool for further real-time applications if the performance in classifying a new animal would confirm the results obtained in the presented study. Additionally, a future algorithm could take care of misclassifications by observing consequent time windowed dataset classification output before determining the actual class, evaluating the class in majority. This strategy would be similar to the one implemented in several implantable devices such as pacemakers (e.g., Amplia MRI™ Quad CRT-D SureScan™, Medtronic, USA). This secondary check could help avoid misclassifications and, consequentially, non-necessary stimulations.

The time interval resulting from time window analysis evaluation in terms of mean test accuracy between the animals could be compatible with online windowing and consequent classification. These results would incite the development of a real-time closed-loop system for simultaneous classification and stimulation of the pudendal nerve for the treatment of LUTD. Computational performance appeared to be compatible with real-time applications, with an observed mean time of 16.2 ms between feature extraction and model prediction for a single sample, corresponding to each time frame.

This study has some limitations. In particular, the results were obtained from a limited number of animals, with a limited number of classes considered. Hence, further investigation should be done. Similarities between target animal and human anatomy could facilitate the possible translation of the presented procedure and analysis to the clinic. Nevertheless, experimental translation to humans still needs to be investigated.

Even though the results of computational performance and optimal time window obtained from the corresponding analysis should be compatible with real-time applications, online recording, and consequential decoding still needs to be tested. Future work will be dedicated to the deployment and consequent testing of the presented algorithm in customized hardware in substitution of the TDT system for online decoding during *in vivo* tests.

Considering the promising results obtained from the MLP model classification, additional multi-animal model training could be further investigated through deep learning algorithms. Hence, the presented results may be implemented in real-time applications for appropriate conditional stimulation of the pudendal nerve for LUTD treatment stimulation based on the classification of the signal recorded via the intrafascicular interface.

## IV. CONCLUSIONS

In this study, intraneural signals were recorded from the pudendal nerve of anesthetized pigs during *in vivo* experiments, with the aim of decoding neural information related to bladder fullness. We developed an ENG decoding algorithm for bladder state detection, constituting a promising tool for extracting information from the pudendal intrafascicular signal in real-time. This leads to the possibility of implementing a closed-loop stimulation protocol based on online bladder information.

Further studies involving pudendal nerve signal analysis related to LUTD will take advantage of this strategy focusing on the implementation of a closed-loop setup in future animal studies, assisting the correct micturition act while restoring natural bladder control and sensation.

## V. METHODS

### A. Intrafascicular electrode design specifications

The design of intrafascicular electrodes used for the experimental data acquisition described in Sec. V B was inspired by TIME design,^26^ as a good trade-off in terms of stability and selectivity with proven long-term efficacy.^18^ In fact, tined lead electrodes used in clinical practice can move from the implant site, losing effectiveness in the long term,^11^ and show low selectivity in fascicle recruitment,^18^ causing side effects such as pain or toe dorsiflexion.^11,18^ Highly selective Utah slanted electrode arrays within the pudendal nerve in cats^22^ implants demonstrated a high recording efficiency, yet their implantation implies a hazardous level of invasiveness in the long-term.

The electrodes consisted of a polyimide intrafascicular electrode with platinum AS coated with iridium oxide (IrOx). We derived anatomical characteristics of the porcine pudendal nerve to tailor the electrode design to target as many fascicles as possible maximizing neural selectivity.

A total of eight pudendal nerve samples were explanted from five farm pigs (*Sus scrofa domesticus*, 3 months of age, 30 kg weight) by using the surgical approach described in Sec. V B. For three out of five of the animals, both the right and left pudendal nerves were explanted. Two centimeters long pudendal nerve samples were fixed in buffered 10% formalin (Bio-Optica, ITA), dehydrated, and embedded in paraffin wax.

Samples were cut with a microtome (Leica RM 2055) into 5 *μ*m thick sections 100 *μ*m apart and stained following Mallory's trichrome protocol.

Microphotographs were acquired under a Nikon Ni-e light microscope (Nikon Instruments Spa, Calenzano, ITA), with a 4× objective, connected to a personal computer via a Nikon digital image processing software (Digital Sight DS-U1, NIS-Elements BR-4.51.00 software).

Two sections per nerve around 1 cm apart for a total of 16 sections were selected and analyzed (Fig. 2). For each section, the maximum and minimum diameter of the nerve, the number of fascicles, and the diameter of the smaller fascicle were estimated using the open-source software Fiji (ImageJ, National Institutes of Health, US). The maximum and minimum diameters of the nerve were used to evaluate the mean nerve diameter for each section. The average and standard deviation were calculated for each of the parameters.

The final electrode design consisted of a total of 16 round ASs (ø = 60 *μ*m) distributed eight per each side of the sandwich structure of the TIMEs. AS diameter was chosen according to the smallest fascicle diameter. The inter-AS distance was set to 0.134 *μ*m to have a maximum of 1 mm distance between most distant ASs, as the nerve mean diameter. The electrode width at the ASs level was 545 *μ*m.

The fabrication process to realize the 10 *μ*m thick polyimide sandwich structure followed standard photolithographic techniques.^26,27^ A 4-in. silicon wafer was coated first with a sacrificial layer of Ti/Al (20/100 nm) using an e-beam evaporation technique then 5 *μ*m of polyimide (PI2611, HD Microsystems GmbH, GER) were spin-coated and cured for 2 h at 300 °C in a N_2_ hard-baking oven. A 4 *μ*m-thick photoresist (ECI 3027, MicroChemicals, GER) was spin-coated, baked, exposed, and developed on top of the previously described layer. According to Cogan and Boehler *et al.*,^28^ Ti/Pt/IrOx (25/300/400 nm) was sputtered (AC450, Alliance Concept) and patterned through liftoff.^24^ Encapsulation of tracks was performed with 5 *μ*m-thick layers of polyimide spin-coated and cured on previous layers. A 12 *μ*m positive photoresist (AZ10XT, MicroChemicals, GER) was spin-coated, baked, exposed, and developed. Shaping of the electrode and exposure of ASs and contact pads were obtained by O_2_-based reactive ion etching (CORIAL 210IL, Plasma-Therm, FL). Anodic dissolution of aluminum in a 1.5 V bias in saturated NaCl solution was used to release the devices.

The two ends of the electrodes were fixed with silver conductive epoxy (Ablestick 84–1LMI, Loctite, GER) on two custom-designed Printed Circuit Boards (PCBs) (Cad Line, ITA) with eight pads, respectively. The two PCBs were bonded together with ultraviolet curable glue (1401-M-UR, Dy- max, GER) to have a 10 *μ*m-thick polyimide sandwich structure, creating a loop in which a suture needle (10–0 Prolene, Johnson & Johnson, USA) was threaded.

Two 16 ASs TIMEs were manually soldered with copper wires on a 32-pin connector (A79022–001, Omnetics Connector Corporation, USA).

### B. Experimental setup and data acquisition

The study was conducted on five castrated male farm pigs (*Sus Scrofa Domesticus*) of age 3–4 months with weights of 30–32 kg.

The animals were premedicated with Zoletil^®^ (10 mg/kg). Next, the animals were anesthetized using Propofol (2 mg/kg intravenously) and maintained under 1%–3% sevoflurane in air enriched with 50% oxygen. The oxygen saturation, arterial pressure, and heart rate were constantly monitored.

Left pudendal nerve was surgically exposed by using the transgluteal approach described in our work conducted on pigs.^28^ Briefly, the animal was placed in the prone position [Fig. 5(a)]. A cutaneous incision was made to expose the gluteal fascia and thus access the space between the superficial and middle gluteal muscles, which were retracted. Pudendal nerve was identified as medially lining the internal pudendal artery and vein [Fig. 5(b)].

The nerve trunk was verified via whole-nerve stimulation by using a custom hook electrode (stimulus frequency = 3 Hz, stimulus amplitude = 1–2 mA). External anal sphincter contraction confirmed pudendal nerve identification.

For all the animals, the left pudendal nerve was implanted with two TIMEs endowed with 16 ASs, spaced 5 mm apart [Figs. 5(c)]. Silicone biocompatible glue (Kwick-Sil, World Precision Instrument LLC, USA) was applied to the implanted electrodes to fix the position [Figs. 5(d)]. The wound was closed and sutured to prevent TIMEs displacement.

The animal was then turned over in a supine position. A midline incision of the abdomen was performed to expose the bladder. A first 16 Fr Foley catheter was inserted and secured within the bladder using a Tobacco-pouch suture technique. The catheter was then connected to a syringe pump (New Era Pump Systems, Inc., Farmingdale, NY) holding a maximum volume of 150 ml of solution. The pump was used to infuse the bladder with saline solution. A second 12 Fr Foley catheter was inserted caudally into the previous one and secured within the bladder with the same surgical technique. This catheter was used to measure bladder pressure. The abdomen was closed and sutured to prevent visceral organ displacement and restore natural pressure inside the abdominal cavity. Finally, a rectal catheter was inserted within the rectum to measure the abdominal pressure.

The two 16 ASs TIMEs, implanted in the pudendal nerve and previously soldered to a 32-pin connector as described in Sec. V A, were connected to the pre-amplifier (Pz5, Tucker-David Technologies Inc., TDT, USA) by using a Hirose connector (DF30FC-40DS-0.4V, Hirose Electric, USA) with an external tungsten needle soldered. The needle was placed within the wound as a ground electrode. The RZ5D BioAmp Processor (Tucker-David Technologies Inc., TDT, USA) acquired the pre-amplified neural data as well as the physiological parameters (intravesical pressure, rectal pressure, and infused volume) using the analog-to-digital channels.

A custom code was implemented in Synapse software (Tucker-David Technologies Inc., TDT, USA) to define data acquisition parameters: ENG signals were sampled at a frequency equal to 24414 Hz while physiological parameters were sampled at a frequency equal to 1526 Hz.

Before starting each experimental session, the bladder was emptied. The intravesical pressure at the beginning of the experiment was around 2 mm Hg.

The impedance of all 32 channels of TIMEs was measured after implanting the electrodes and compared with the same measurement performed in saline solution immediately before implanting the electrode to verify the proper functioning and electrical conductivity of the electrodes.

The number of nonfunctioning electrodes was found to be between 4 and 7 (the latter in only two cases). In each case, all channels were considered for analysis, even those that did not work, in order to simulate the worst case and thus improve the robustness of the outcome. The neural peripheral activity was recorded by using the electrodes implanted within the pudendal nerve during a baseline condition and functional urinary challenge that modifies physiological parameters, such as intravesical pressure. Baseline condition was defined as a period in which intravesical pressure was stable for at least 5 minutes after electrode insertion. The functional urinary challenge consisted of subsequent steps of saline solution infusion within the bladder (Fig. 1). Each step consisted of 5 minutes of registration distributed as follows: 1 minute of data recording before starting with the infusion, 3 minutes for the infusion of 150 ml of saline solution with 50 ml/min flow rate, and 1 minute of data recording after infusion. This protocol was repeated until urine leakage was visible. When a transurethral leakage occurred, the pump was stopped and a full bladder recording session lasting 5 minutes was acquired. Then, the last infusion session was performed as described before to acquire data during urine leakage.

### C. Data description, preprocessing, time framing, and feature extraction

The coding strategy was developed by adapting the algorithm presented in the previous work from our group for vagus nerve classification during cardiac and respiratory challenges.^20^ For each animal, a dataset constituted by ENG signals obtained from the three state recordings was created and ordered. Sessions indicated as Micturition were extrapolated for the micturition time interval of interest. All the sessions were cut at the same duration in order to avoid class unbalance. For all sessions, all 32 ASs raw ENG signals were imported into the structure.

**FIG. 9.**
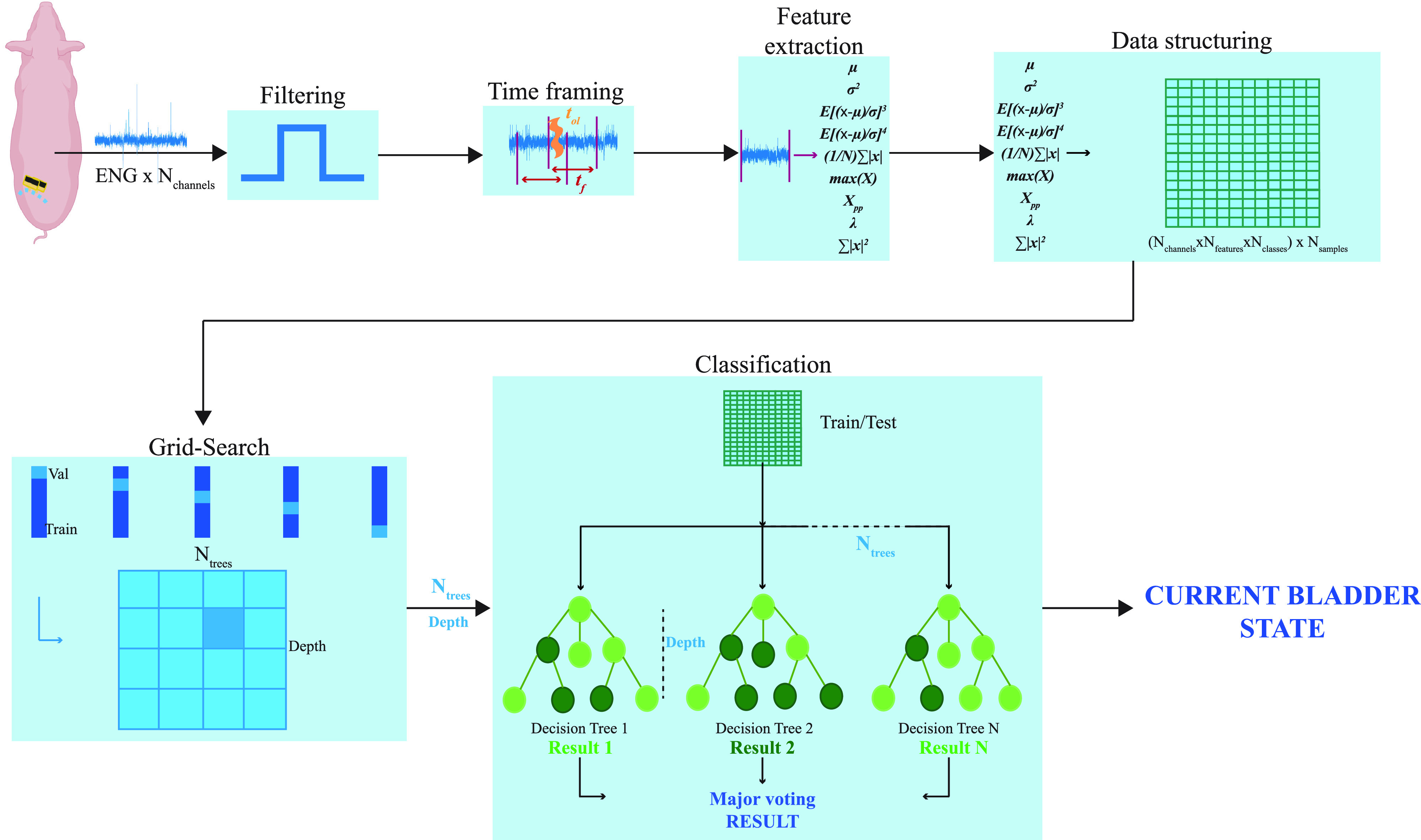
ENG signals decoding process from filtering to classification. 32-channel × N_classes_ are filtered and structured in order to perform model optimization and final prediction based on *BA* computation.

The following ENG signal processing steps were illustrated in Fig. 9. Single-channel recordings were independently filtered using a Butterworth Band Pass Filter of fourth order with cutoff frequencies equal to f_l_ = 1 kHz and f_h_ = 6 kHz. The choice was based on the evidence of a particular localization of spikes of somatic nerve fibers.^29^ After filtering, all signals were undersampled by a factor of 2.

Data were split into testing and training sets with a ratio equal to 1:10. Single-channel ENG recordings of the training set were independently subdivided by using the time windowing, consisting of partitioning the signal in equal length sub-windows with an overlap equal to 20% between contiguous windows. The test set was windowed using the same window length without overlapping. Each time sample obtained was transformed into nine handcrafted features, characterizing the distribution of electrical potential in the different samples.^20^

**FIG. 10.**
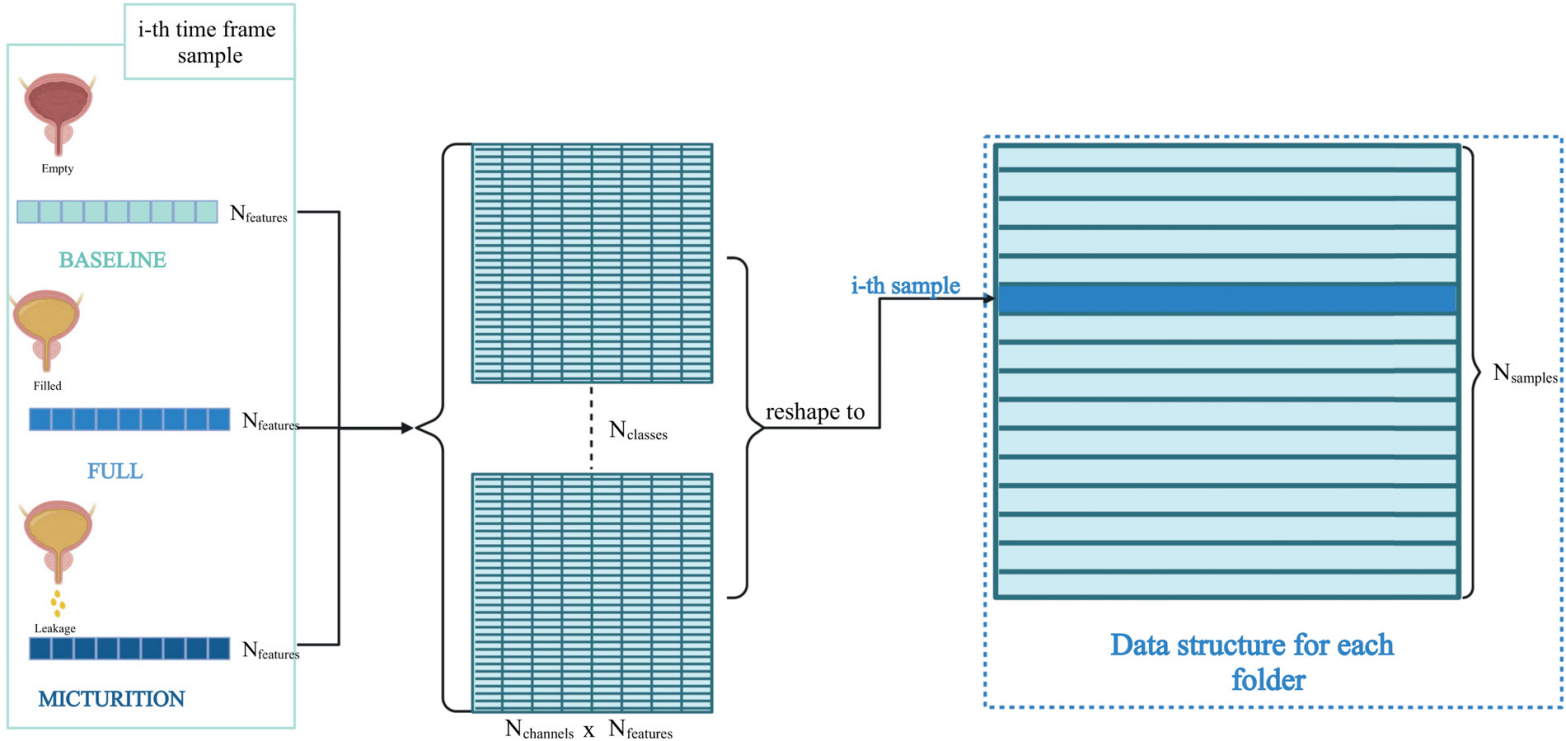
ENG signals features from each class of the ith time frame concatenation and structuring.

Feature extraction was performed independently for each time frame of the ENG signal and AS. The chosen features were mean, variance, skewness, kurtosis, maximum amplitude, wavelength, maximum, amplitude, and power. Then, for a single time frame, features' matrices of each channel were concatenated together. Finally, data were reconstructed by arranging the concatenated features' matrices relative to each time frame in columns (Fig. 10).

Training dataset was then standardized along features' dimensions by removing the mean and scaling to standard deviation. Test set was scaled with reference mean and variance values computed from the training set. Sample clustering was avoided by shuffling their order for each set.

### D. Machine Learning models

Four classifiers were tested for our goal: SVM, k-NN, RF, and MLP.

SVM algorithm was widely implemented in various applications involving ENG signal classification, such as sensory information decoding,^30,31^ and closed-loop applications,^32^ showing valid results. The k-NN algorithm was taken into account for the final model classifier choice since used successfully in upper-limb amputees ENG closed-loop systems, revealing satisfactory results in online decoding.^19^ The RF and MLP models constituted, based on motor intention recognition from peripheral nerves in amputees, a possible alternative to DL models in terms of computational cost and performance.^33^

The first animal in the analysis was chosen to compare the different models' performances. The models were compared in terms of test *BA* performance. The RF model, in particular, showed a lower chance of overfitting with higher performance, when compared to more complex models, such as neural networks.

### E. Model optimization

In order to find the best set of hyperparameters for each model, Grid-Search was performed during the Nested-Cross Validation (NCV) phase. The training set was divided between the validation and the inner training folder. Thereby, inner loops were performed, shifting the validation folder along the time dimension at each loop. For each round, Grid Search was performed: models were fit and evaluated, respectively, on training and validation sets, considering all possible combinations of hyperparameters.

The optimal combination was chosen on a model performance basis and consequently applied to the final model configuration. Optimized model design was then trained and tested.

Different hyperparameter values were tested for each model. The following number of trees [50, 100, 200, 300] and depth [5, 10, 20, 30] values for the RF have been chosen, while the SVM kernel function was set as a sigmoid or radial basis function, while L2 regularization value was tuned between the following values: 10–3, 10–2, 10–1, 1. In k-NN, the number of neighbors varied between 3, 5, 7, 9, and 11; while for the MLP five neural networks were tested, with the following number of neurons for each layer: [128, 128, 128, 128] [128, 256, 256, 128], and [128, 256, 512, 128] [128, 512, 512, 128] [128, 256, 512, 512], imposing a learning rate equal to 10^−3^.

A MacBook Pro 13-in., 2016, Apple Inc., with a 2.9 GHz dual-core Intel Core i5 processor and 16 GB RAM memory was used for performing the overall data processing. Initial importing code for TDT system acquired data conversion was written in MATLAB (MathWorks, USA), while preprocessing, classification, model comparison, and plotting codes were written in Python, using the following libraries: Scikit-learn, Pandas, and PyTorch.

### F. Time window analysis

Frame window analysis was performed in order to select a time window length suitable for real-time adequate performance and proper feature computation. All animals' classification through the RF model was performed, varying time window length and overlap. The time window length values tested were twenty, in the range from 50 ms to 1 s. The overlap was always equal to 20% of the picked time window length. The mean *BA* trend along all time window values in exam was observed by comparing all animals' results. A trade-off between time lengths corresponding to the highest *BA* results obtained between the pigs was chosen.

### G. Performance evaluation

Time window length selection was performed to evaluate the model performance when applied 20-time frame values in a range between 0.05 and 1 s for all animals' ENG classification. Model performance has been evaluated by computing the *BA* score.

*BA* was computed as follows:

$$BA={\text{Recall}}_{B}+{\text{Recall}}_{F}+{\text{Recall}}_{M},$$

where Recall_*n*_ represented the sum of True Positives (TP) across the *N*th class, divided by the sum of all TP and False Negatives (FN) relative to the class. When RF was implemented, feature importance was evaluated through the tree impurity quantification by the Gini importance index for split quality and square root function for deciding the maximum number of features destined for each tree. Single AS weight and average feature decrease in impurity were obtained, allowing the observation of ASs' effective contribution to the final *BA* and feature importance across all animals.

## SUPPLEMENTARY MATERIAL

See the supplementary material for details on histological images of porcine pudendal nerve, pudendal nerve electroneurographic signals, and associated detected spikes.

## Data Availability

The data that support the findings of this study are available from the corresponding author upon reasonable request.
